# The Dirac Equation, Mass and Arithmetic by Permutations of Automaton States

**DOI:** 10.3390/e27040395

**Published:** 2025-04-07

**Authors:** Hans-Thomas Elze

**Affiliations:** Dipartimento di Fisica “Enrico Fermi”, Universitá di Pisa, Largo Pontecorvo 3, 56127 Pisa, Italy; elze@df.unipi.it

**Keywords:** Dirac equation, Weyl equation, cellular automaton, permutations, Ising spin, qubit, ontological state, superposition principle, quantum mechanics

## Abstract

The cornerstones of the Cellular Automaton Interpretation of Quantum Mechanics are its underlying ontological states that evolve by permutations. They do not create would-be quantum mechanical superposition states. We review this with a classical automaton consisting of an Ising spin chain which is then related to the Weyl equation in the continuum limit. Based on this and generalizing, we construct a new “Necklace of Necklaces” automaton with a torus-like topology that lends itself to represent the Dirac equation in 1 + 1 dimensions. Special attention has to be paid to its mass term, which necessitates this enlarged structure and a particular scattering operator contributing to the step-wise updates of the automaton. As discussed earlier, such deterministic models of discrete spins or bits unavoidably become quantum mechanical, when only slightly deformed.

## 1. Introduction

This work is motivated by an hypothetical deterministic ontology to understand natural phenomena which are studied in physics and chemistry and possibly beyond. This might contrast with the perception of an intrinsic fundamental randomness especially in the dynamics of microscopic objects, such as Standard Model ‘elementary’ particles, atoms, or molecules, which is described, for example, in the references [1,2,3]. Related ideas are obviously founded on very successful developments and applications of quantum theory in the study and experimental manipulation of the microscopic world.

Here, we do *not* base our discussion on quantum theory. Yet, we will quickly find it convenient to borrow elements from its most efficient language and formalism. Our working hypothesis is what we observe in experiments, we ‘see’ in terms of descriptions employing the language of mathematics and especially of quantum theory.

We follow the *Cellular Automaton Interpretation of Quantum Mechanics*, namely that quantum mechanical features can be found in the behavior of certain classical cellular automata [4,5]. Thus, deterministic dynamics is considered to underlie any change occurring with an object, i.e., a change of its ontological state.

The universe is always in a specific, even if unknown to us, ontological state. In the ‘toy models’ presented in the following, the model universe consists of a countable number of two-state Ising spins that evolve according to deterministic automaton rules. Incidentally, the yes–no or spin-up–spin-down alternatives here may be described as occupation numbers of fermionic degrees of freedom, i.e., with values 0 or 1. This possibility has been explored in quantum field theories of fermions built from classical *probabilistic cellular automata*, e.g., in reference [6].

We shall stick to strictly deterministic evolutions of cellular automata here, as in earlier work [7], and will not discuss anew arguments for and against attempts to reinstall determinism at a fundamental level of natural science. Similarly, we refer the reader to the literature concerning the measurement problem of quantum theory, which in an ontological theory does not exist [4], and the violation of Bell’s inequality, which does not necessarily rule out such a theory [4,8,9,10,11].

These considerations invoke, in one way or another, a *superdeterministic* picture of the universe. This is most clearly visible in ’t Hooft’s explanation of the non-classical correlations, experimentally observed as violations of Bell’s inequality, by vacuum correlations established in the past of such an experiment [4]. However, the notion of vacuum correlations has a precise meaning only in *quantum* many-body or field theory and, therefore, it seems plausible but not convincing to invoke this for the understanding of such an essential effect from an ontological perspective. There is no ontological model yet that produces deterministically such quantum fluctuations. However, as far as deterministic ontological models, such as the Cogwheel Model (Appendix A.1), have a quantum mechanical limit or become quantum mechanical “by mistake” (cf. below); however, they do also open an avenue to understand quantum fluctuations and correlations. Clearly, this remains to be explored and is not our aim here. – An interesting probabilistic approach is presented by Wetterich that bridges between classical determinism (such as underlying classical statistical mechanics) and quantum mechanics (possibly arising by evolution from certain probabilistic initial conditions for otherwise deterministic dynamics) [12].

Instead, we shall discuss interesting features of ontological states realized in classical Ising models which evolve deterministically through permutations.

We remark that there are *no superposition states* created by any dynamics based on permutations. Superpositions do not exist in a set of ontological states; yet, they are conveniently introduced in our mathematical language, the *quantum superpositions* of ontological (micro) states, which do not exist ‘out there’ as states the universe could be in. On the other hand, *classical states* are ontological (macro) states, or probabilistic superpositions thereof, appropriate for nature’s phenomena at vastly different scales [4]. – The question of practical as well as foundational interest, whether there is a consistent notion of quantum-classical hybrids (cf. [13]), has been asked in the present context with a negative answer [14].

We do not aim to replace quantum theory as the physicist’s perfect toolbox. However, a better understanding of its peculiarities is desirable, just as it is desirable to recover the structural elements of its mathematical language. Our approach should be seen as an attempt in this direction, based on the assumption of a deterministic ontology and realized in terms of classical two-state Ising spins or bits. This is distinctly different technically and conceptually, but similar in spirit to other more or less evolved, though critically discussed approaches to “interpret” quantum theory. – For example, Bohmian mechanics [15,16,17], stochastic electrodynamics [18,19,20], prequantum classical statistical field theory [21], or quantum Bayesianism (*QBism*) [22] all study the emergence, rewriting, or generalization of quantum theory from various perspectives. However, none of the latter interpretations really go outside of quantum theory. They are occupied with not getting into conflict with its perfectly working theoretical “machinery” while trying to add elements helping its understanding. – Thus, Bohmian mechanics *adds* to the continuum theory of Schrödinger’s equation for the wave function apparently classical trajectories of point particles, which are guided by the wave function. An ontology that would complete this interpretation and allow us to understand the where-from and what-for of the wave function is missing. On the other hand, *QBism* does *not* seem to address at all the “stuff” we perceive “out there” and describe in physics. It rather tries to make comprehensible a central aspect of quantum theory, its probabilistic character with Born’s rule, by relating it to rationally acting agents that can make predictions, verify them, and improve prior beliefs. – These interpretations, and likewise others not mentioned here, seem far from the program advocated by ’t Hooft and followed in a specific way in this article.

Before introducing more detailed models in this paper, it may be worthwhile to recall that models based on permutations of ontological states lead to Hamiltonians which are rather poor in tunable parameters or coupling constants. In the long run of developing a future ontological theory, this may be a desirable feature.

Here, it suffices to point out that a Hamiltonian pertaining to the unitary dynamics generated by permutations will be completely fixed, once an underlying Ising model and its updating rule and time step size are defined. However, with respect to realistic circumstances, trying to pin down a theoretical model based on experimental data, inaccuracies of extracted parameters cannot be avoided. Such unavoidable imprecision will replace the ‘true’ Hamiltonian $\hat{H}$ – describing deterministic evolution of ontological states – by a result based on experiments, ${\hat{H}}_{exp}=\hat{H}+\delta \hat{H}$. And most likely ${\hat{H}}_{exp}$ will generate superposition states, which leads necessarily to a Hilbert space description. This indicates that the classical automaton models considered here are always intrinsically and unavoidably close to becoming quantum mechanical “by mistake” [23]. It suggests that quantum theory more generally might be a *best possible theory* reflecting our persisting ignorance of the ontology beneath.

Ingredients for our present considerations were earlier obtained and reported in references [23,24]. In particular, in [24] we presented further motivation and context of attempts to find an ontological basis for quantum theory, including additional references. In order to make this article self-contained, we summarize some of the formal aspects in the Appendix A, while new results are described in the following Section(s).

It is important to realize that the present study can be considered independently of the background of discussions concerning the foundations of quantum theory. In the following derivations, one may consider the Dirac equation as a “classical” field equation, completely independent of its famous quantum theoretical context. And the question raised and answered affirmatively in this work is can we construct a discrete deterministic cellular automaton that captures the dynamics embodied in this equation?

## 2. Results: An Automaton Beneath the Dirac Equation

To set the stage we first explain how the *Weyl equation* for massless spin-1/2 particles is obtained from the cellular automaton described in Appendix A.2, based on the *Cogwheel Model* reviewed in Appendix A.1. This will be followed by the construction of an automaton representing the Dirac equation for massive spin-1/2 particles in 1 + 1 dimensions, which requires additional structure to incorporate the mass.

### 2.1. The Weyl Equation

In order to arrive at the Weyl equation, a continuum field equation, from the discrete cellular automaton based on two-state Ising spins–equivalently, on block numbers defined in Appendix A.2, Equations (A8) and (A9) – we suitably denote an arbitrary state |ψ〉 of the spin chain by

(1) $$\begin{array}{ccc} |\psi \rangle & := & |\{s(2k-1)\},\{s(2k)\}\rangle , \end{array}$$

(2) $$\begin{array}{ccc} & =: & |\left\{s^{L}\left(k\right)\right\},\left\{s^{R}\left(k\right)\right\}\rangle , \end{array}$$

for $s\left(k\right):=s_{k}$, in terms of the spin variable at site *k*, k=1,…,S+1, and with periodic boundary conditions, s(2S+1)≡s(1), s(2S+2)≡s(2). Here, we first group the left- and right-moving spin variables together, and then rename them $s^{L}$ and $s^{R}$, respectively, and renumber the even- and odd-numbered sites.

Considering, for example, the evolution generated by $\hat{U}$ of Equation (A7) in *l* steps, the update of a generic state $|\psi_{n}\rangle$ at time t=nT is exactly described by a pair of equations:

(3) $$\begin{array}{ccc} s_{n+l}^{L}\left(k\right)-s_{n}^{L}\left(k\right) & = & s_{n}^{L}\left(k+l\right)-s_{n}^{L}\left(k\right), \end{array}$$

(4) $$\begin{array}{ccc} s_{n+l}^{R}\left(k\right)-s_{n}^{R}\left(k\right) & = & s_{n}^{R}\left(k-l\right)-s_{n}^{R}\left(k\right), \end{array}$$

where suitable identical terms were subtracted on both sides of Equations [23,24].

Then, for *l* = 1, Equations (3) and (4) simply represent discretized first-order partial differential equations in 1 + 1 dimensions. Remarkably, the left- and right-movers do not interact and up-and-down spins are never flipped here. – Identical equations hold for the block numbers introduced in Equation (A9) in Appendix A.2.

Jumping to the continuum limit, we introduce linear combinations $S^{\pm }:=s^{L}\pm s^{R}$ and ${\bar{\sigma }}^{0}:=1_{2}$, ${\bar{\sigma }}^{1}:=-\sigma_{x}$ with unit matrix $1_{2}$ and Pauli matrix $\sigma_{x}$. Thus, the Equations (3) and (4) can be combined and yield the *left-handed Weyl equation*:

(5) $${\bar{\sigma }}^{\mu }\partial_{\mu }\Psi_{L}=\left(1_{2}\partial_{t}-\sigma_{x}\partial_{x}\right)\Psi_{L}=0,$$

for the two-component ‘spinor’ ${\left(\Psi_{L}\right)}^{t}:=\left(S^{+},S^{-}\right)$—the *right-handed Weyl equation*, with $\sigma^{0}:=1_{2}$, $\sigma^{1}:=+\sigma_{x}$, and ${\left(\Psi_{R}\right)}^{t}:=\left(S^{+},S^{-}\right)$,

(6) $$\sigma^{\mu }\partial_{\mu }\Psi_{R}=\left(1_{2}\partial_{t}+\sigma_{x}\partial_{x}\right)\Psi_{R}=0,$$

is obtained similarly, especially replacing $\partial_{x}\to -\partial_{x}$ in the derivation following Equations (3) and (4), which amounts to an additional overall minus sign on the right-hand sides of both equations or exchanging left- and right-movers there. In turn, this is equivalent to numbering the sites of the spin chain from right to left instead of from left to right, as before.

The continuum limit can be studied in a controlled way, for example, by applying *Sampling Theory*, similarly as in references [7,25,26,27]. This maps the above *finite differences equations* one-to-one on continuous space-time partial differential equations for *bandwidth-limited fields* $s^{L}\left(x,t\right)$ and $s^{R}\left(x,t\right)$, i.e., continuum equations for functions with an *ultraviolet cut-off* corresponding to a nonzero scale *T*. There arise higher-order derivative terms from expanding $\exp(T\partial_{t})$ or $\exp(T\partial_{x})$ (c=1), which disappear for T→0. – Note that on discrete lattice points, the functions $s^{L,R}\left(x,t\right)$ can only assume the Ising spin values $s_{k}=\pm 1$, while they can be real-valued in between. This limitation is overcome by assuming block numbers as underlying discrete variables which, by construction, range over a larger set of non-negative integers; see Appendix A.2.

Obviously, there are no mass terms in the Weyl equation. However, in reference [24], we showed that the implied signal velocity can be changed to differ from c=1 by modifying the update rule in simple ways. Nevertheless, this does not correspond to introducing a mass, as will be needed for the Dirac equation, cf. the following Section 2.2.

### 2.2. Reverse Engineering an Automaton Based on Permutations from the Dirac Equation

We have studied the deterministic cellular automaton based on an Ising spin chain with exchange interactions, i.e., transpositions of two-state variables. This has led to the Weyl equation for *massless* particles in the continuum limit, but it did not provide insight into how to generate the mass terms for the Dirac equation. In order to proceed, we elaborate in the following the *“reverse engineering”* approach suggested previously [24].

Consider the real representation of the Dirac equation in 1 + 1 dimensions [28]:

(7) $$\partial_{t}\Psi =\left(\sigma_{z}\partial_{x}-i\mu \sigma_{y}\right)\Psi ,$$

where $\Psi^{t}:=\left(\Psi_{1},\Psi_{2}\right)$ is the two-component real spinor field, $\sigma_{y,z}$ are the usual Pauli matrices, and μ denotes a dimensionless mass parameter; here, we choose units such that c=ℏ/m=1 for the dimensional mass scale *m*. – The Equation (7) is equivalent to two coupled partial differential equations:

(8) $$\begin{array}{ccc} \partial_{t}\Psi_{1} & = & \partial_{x}\Psi_{1}-\mu \Psi_{2}, \end{array}$$

(9) $$\begin{array}{ccc} \;\;\;\;\partial_{t}\Psi_{2} & = & -\partial_{x}\Psi_{2}+\mu \Psi_{1}. \end{array}$$

Our strategy now is to discretize them, going backward in the direction of Equations (1)–(4), and see the changes brought about by the presence of mass terms.

**Figure A2:**
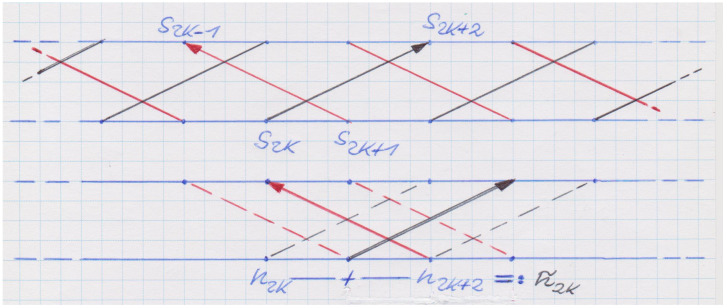
(**Top**): Spin variables on *odd/even* sites are transported to the next *odd/even* sites to the *left/right*, respectively, in one update by $\hat{U}$, Equation (A7), of the spin chain automaton. (**Bottom**): Occupation numbers on neighboring even sites are added to form right-moving block variables, $n_{2k}+n_{2k+2}=:{\tilde{n}}_{2k}$; likewise, left-moving ones, $n_{2k+1}+n_{2k+3}=:{\tilde{n}}_{2k+1}$. They are illustrated by arrows displaced by one unit to the right for better visibility.

We replace $\Psi_{1}\left(x,t\right)\to s_{n}^{L}\left(k\right)$ and $\Psi_{2}\left(x,t\right)\to s_{n}^{R}\left(k\right)$, i.e., directly in terms of what have been left- and right-movers, respectively. Thus, instead of Equations (3) and (4), we obtain here

(10) $$\begin{array}{ccc} s_{n+1}^{L}\left(2k-1\right) & = & s_{n}^{L}\left(2k+1\right)-\mu s_{n}^{R}\left(2k\right), \end{array}$$

(11) $$\begin{array}{ccc} \;\;\;\;\;\;\;\;\;\;\;\;\;\;s_{n+1}^{R}\left(2k\right) & = & s_{n}^{R}\left(2k-2\right)+\mu s_{n}^{L}\left(2k-1\right), \end{array}$$

for k=1,…,S+1, with periodic boundary conditions, and canceling identical terms on both sides of the equations that arise from the discretization of the derivatives. Herein, the notation is as earlier in Equation (1), yet we left the superscripts .L,R for clarity: the left-movers (right-movers) occupy the odd (even)-numbered sites of a chain, cf. Figure A2 (top).

From the Equations (10) and (11), one can derive second-order finite difference equations. They agree with those obtained directly by discretizing the second-order wave equations for the spinor components in the continuum. Equations (10) and (11) can also be inverted in order to obtain the automaton equations for the deterministic evolution backwards, determining the earlier $s_{n}^{L,R}$ in terms of the later $s_{n+1}^{L,R}$.

Note that the mass terms add simple inhomogeneities to the previous equations for left- and right-movers. Therefore, the variables can no longer be related to *two-state* Ising spins as before, cf. Section 2.1. However, we set μ=1 henceforth, such that they may still be considered integer valued. – This could be consistent with an underlying Ising spin chain if we repeatedly perform the transformation on Equations (10) and (11) that we discuss in Appendix A.2, following Equations (A8) and (A9). It would lead to possibly large *block variables* instead of the *two-state* Ising spins. However, it turns out that the attempt to construct an automaton in the way sketched in [24] does not succeed and the set-up has to be changed more profoundly. This will be shown in the following Section 2.2.1.

#### 2.2.1. Doing Arithmetic by Permutations

Our aim is to handle also the additive contributions produced by the mass terms by *permutations*, besides those that produce the left- and right-moving kinematics in the above equations, as we have seen earlier. In this way, the update rule of the new cellular automaton will altogether be described by permutations, extending the *Cellular Automaton Interpretation* from the case of the Weyl equation (Section 2.1) to the Dirac equation.

However, as indicated above, we need to generalize the model of the Ising spin chain in order to accommodate the arithmetic implied by the mass terms of the Dirac equation. Instead of an Ising spin chain with periodic boundary conditions, we will implement a *“Necklace of Necklaces”*. This we already alluded to in reference [24]. However, its torus-like topology will now be made use of differently, once again based on *two-state Ising spins*.

Borrowing notation from quantum theory, the state of the automaton at time t = nT is represented by

(12) $${|\Psi \rangle }_{n}:=|s_{1,\;l_{1}},\;\ldots ,\;s_{2k,\;l_{2k}},\;\ldots ,\;s_{2k+1,\;l_{2k+1}},\;\ldots ,\;s_{2S,\;l_{2S}}\rangle ,$$

where the spin variables can assume values s=±1. We note that the variables here are labeled by *two indices*, an either even or odd index for the “longitudinal” position, with k=1,…,S , and an index for the “transverse” position, $-S,-S+1,\ldots ,l_{k},\ldots ,S-1,S$; the index *k* on $l_{k}$ will mostly be suppressed when no confusion arises.

Furthermore, the following torus-like *periodic boundary conditions* are assumed:

(13) $$\begin{array}{c} s_{2S+1,\;l}=s_{1,\;l},\;\;s_{2S+2,\;l}=s_{2,\;l},\;\;\mathrm{for}\mathrm{all}\;l, \end{array}$$

(14) $$\begin{array}{c} \;\;\;\;\;\;\;s_{k,\;S+1}=s_{k,\;-S},\;\;s_{k,\;-S-1}=s_{k,\;S},\;\;\mathrm{for}\mathrm{all}\;k. \end{array}$$

Thus, the index *k* labels even- and odd-numbered sites along a *“necklace”* in the longitudinal direction and each such site is part of a transverse *“necklace”*; altogether sites are labelled by $k,l_{k}$, often simplified to k,l, hence the name *“Necklace of Necklaces”*.

The states of the automaton, cf. Equation (12), are further restricted to have, for each longitudinal position, say $k^{\prime }$, *exactly one spin up* with $s_{k^{\prime },\;l^{\prime }}=+1$ and *all other spins down* with value $s_{k^{\prime },\;l\neq l^{\prime }}=-1$.

The operator $\hat{L}\left(k^{\prime }\right)$ which measures the position of the *one up spin* at a given $k^{\prime }$ is then defined by

(15) $$\hat{L}\left(k^{\prime }\right):=\sum_{l=-S}^{S}\frac{l}{2}\left({\hat{\sigma }}_{k^{\prime },\;l}^{z}+1_{2}\right),$$

where ${\hat{\sigma }}_{k^{\prime },\;l}^{z}$ denotes the usual diagonal Pauli matrix acting on the spin $s_{k^{\prime },\;l}$. The restricted automaton states are eigenstates of this operator, such that for $s_{k^{\prime },\;l^{\prime }}=+1,\;s_{k^{\prime },\;l\neq l^{\prime }}=-1$ we obtain $\hat{L}\left(k^{\prime }\right){|\Psi \rangle }_{n}=l^{\prime }{|\Psi \rangle }_{n}$.

We shall now employ the transverse position $l^{\prime }$ of a single up spin with given longitudinal position $k^{\prime }$ to encode the integer value of the variables $s_{n}^{L}\left(k^{\prime }\right)$ ($k^{\prime }$ odd) or $s_{n}^{R}\left(k^{\prime }\right)$ ($k^{\prime }$ even) that are described by the Equations (10) and (11). Additions and subtractions due to the mass terms then correspond to *shifts of up spin positions*.

A *positive unit shift* for a state with a single up spin at $k^{\prime },l^{\prime }$ is effected by moving *all* spins with this $k^{\prime }$ one step towards increasing transverse position $l_{k^{\prime }}$. This is achieved by a complete permutation – see Appendix A.1 – with a *unitary operator* ${\hat{U}}_{⊥}$:

(16) $${\hat{U}}_{⊥}\left(k^{\prime }\right)|\;\ldots ,\;s_{k^{\prime },\;l_{k^{\prime }}},\;\ldots \rangle :=|\;\ldots ,\;s_{k^{\prime },\;l_{k^{\prime }}+1},\;\ldots \rangle ,$$

for all spins at fixed $k^{\prime }$ with l∈[−S,S], respecting the periodic boundary condition. This operator is explicitly given in terms of *pairwise transpositions* of nearest neighbor spins:

(17) $${\hat{U}}_{⊥}\left(k^{\prime }\right):=\prod_{l=-S}^{S}{\hat{P}}_{l\;l+1}\left(k^{\prime }\right),$$

which are discussed in Appendix A.2. Using unitarity, with ${\hat{U}}_{⊥}^{-1}={\hat{U}}_{⊥}^{†}$, a *negative unit shift* can likewise be expressed in terms of elementary transpositions.

It is now clear that additions and subtractions required by the finite difference equations of motion can indeed be performed by suitable (multiple) permutations or transpositions of the encoding Ising spins.

We proceed in several steps. – First, we re-express the right-hand sides of Equations (10) and (11) by corresponding terms using the above restricted Ising spin states and permutations:

(18) $$\begin{array}{ccc} \;\;\;\;s_{n}^{L}\left(2k+1\right)-s_{n}^{R}\left(2k\right) & \to & {\left({\hat{U}}_{⊥}\left(2k+1\right)\right)}^{-s_{n}^{R}\left(2k\right)}{|\Psi \rangle }_{n}, \end{array}$$

(19) $$\begin{array}{ccc} s_{n}^{R}\left(2k\right)+s_{n}^{L}\left(2k+1\right) & \to & {\left({\hat{U}}_{⊥}\left(2k\right)\right)}^{+s_{n}^{L}\left(2k+1\right)}{|\Psi \rangle }_{n}, \end{array}$$

where we shifted k→k+1 in (19), as compared to Equation (11) (with μ=1). The exponents on the right-hand sides of (18) and (19) $\hat{L}\left(k^{\prime }\right)$ defined in Equation (15):

(20) $$\begin{array}{ccc} s_{n}^{R}\left(2k\right){|\Psi \rangle }_{n} & = & \hat{L}\left(2k\right){|\Psi \rangle }_{n}, \end{array}$$

(21) $$\begin{array}{ccc} s_{n}^{L}\left(2k+1\right){|\Psi \rangle }_{n} & = & \hat{L}\left(2k+1\right){|\Psi \rangle }_{n}. \end{array}$$

The updates described in (18)–(21), so far, *cannot* be performed *consecutively*. This would obviously violate the evolution generated by the right-hand sides of Equations (10) and (11).

Instead, they have to take place simultaneously for all positions *k*, mediated locally by a *scattering operator* $\hat{M}\left(k\right)$, which is introduced in

(22) $${|\Psi \rangle }_{n}^{\prime }=\prod_{k=1}^{S}\hat{M}{\left(k\right)|\Psi \rangle }_{n}\;:=\;\prod_{k=1}^{S}\;\;]\begin{array}{c} {\left({\hat{U}}_{⊥}\left(2k\right)\right)}^{+\hat{L}\left(2k+1\right)}\\ {\left({\hat{U}}_{⊥}\left(2k+1\right)\right)}^{-\hat{L}\left(2k\right)} \end{array}[\;{|\Psi \rangle }_{n}.$$

The inverted brackets ]…[ are meant to indicate that the operations listed in between act on *two neighboring transverse necklaces* situated at longitudinal position 2k and 2k+1, respectively, and yield these two necklaces updated according to the right-hand sides of (18) and (19) – Examples illustrating this process are provided in Appendix A.3. – Since operations for one such even–odd position nearest neighbor pair do not interfere with those for another one, the product for all pairs is taken in the end.

We may rewrite the complete permutations implemented by unitaries in Equation (22) as exponentials of the corresponding Hamiltonian, ${\hat{U}}_{⊥}\left(k^{\prime }\right)=\exp\left(-iT{\hat{H}}_{⊥}\left(k^{\prime }\right)\right)$, which has been explicitly given in Appendix A.1; here, it acts on all spins with fixed longitudinal position $k^{\prime }$ and with 2S+1 varying indices for the related transverse necklace. Since, e.g., ${\hat{H}}^{⊥}\left(2k\right)$ and $\hat{L}\left(2k+1\right)$ act on spins with different longitudinal index, they commute and can be exponentiated side by side; also, $[{\hat{H}}^{⊥}\left(2k+1\right),\hat{L}\left(2k\right)]=0$. Thus, we obtain

(23) $$\prod_{k=1}^{S}\hat{M}\left(k\right)\;=\;]\begin{array}{c} \exp\left(-iT\sum_{k=1}^{S}{\hat{H}}_{⊥}\left(2k\right)\hat{L}\left(2k+1\right)\right)\\ \exp\left(+iT\sum_{k=1}^{S}{\hat{H}}_{⊥}\left(2k+1\right)\hat{L}\left(2k\right)\right) \end{array}[,$$

which represents the update operations to be applied on the automaton state ${|\Psi \rangle }_{n}$ to yield the intermediate results corresponding to the right-hand sides of Equations (10) and (11).

Finally, in order to complete the evolution equation for the automaton, we have to take into account that the intermediate state ${|\Psi \rangle }_{n}^{\prime }$ of Equation (22) still has to be modified such that the *left- and right moving kinematics* is incorporated.

According to Equation (10), the result produced by the right-hand side of this equation at longitudinal position 2k+1, especially in absence of a mass term (cf. “Weyl automaton”), has to move *left* by two positions to 2k−1, for all *k*. Similarly, according to Equation (11), the intermediate result at even-numbered longitudinal position 2k has to move right by two positions to 2k+2, for all *k*, cf. Figure A2 (top). These moves complete the *one-step update*:

(24) $${|\Psi \rangle }_{n}\;\overset{{|\Psi \rangle }_{n}^{\prime })}{⟶}\;{|\Psi \rangle }_{n+1},$$

related to the time interval *T*, as before. See also simplest examples in Appendix A.3.

From the discussion in Appendix A.2, we know how left- and right-movers are generated by transpositions acting on a spin chain. Presently, however, we have to move the complete transverse necklaces of spins. This is effected by an obvious generalization of the previous unitary of Equation (A7), i.e., by a unitary operator ${\hat{U}}_{kin}$ defined as:

(25) $${\hat{U}}_{kin}:=\prod_{l=-S}^{S}\prod_{k=1}^{S}{\hat{P}}_{2k-1\;2k;\;l}\prod_{k^{\prime }=1}^{S}{\hat{P}}_{2k^{\prime }\;2k^{\prime }+1;\;l}=:\exp\left(-iT{\hat{H}}_{kin}\right),$$

where the transpositions ${\hat{P}}_{ij;\;l}$ exchange the nearest neighbor spins at longitudinal positions *i* and *j*, respectively, with identical transverse index *l*. Similarly as before, the corresponding Hamiltonian ${\hat{H}}_{kin}$ can be given analytically as a polynomial in ${\hat{U}}_{kin}$ and ${\hat{U}}_{kin}^{†}$ [23].

Thus, we finally arrive at the *one-step update rule* for the cellular automaton reverse-engineered from the Dirac equation in 1 + 1 dimensions:

(26) $${|\Psi \rangle }_{n+1}={\hat{U}}_{kin}\prod_{k=1}^{S}\hat{M}\left(k\right){|\Psi \rangle }_{n}.$$

This presents our main result. – Following the arguments presented in this work, the Equation (26) summarizes the behavior of a new *deterministic cellular automaton* for the Dirac equation in 1+1 dimensions. Emphasis lies on the deterministic evolution, while earlier attempts have been probabilistic or fully quantum mechanical instead.

## 3. Discussion

We emphasize that the dynamics of the *“Dirac automaton”* described above is completely based on permutations of underlying Ising spins, the two-state variables that define its ontological states, in accordance with the *Cellular Automaton Interpretation of Quantum Mechanics* [4].

We had to introduce the *scattering operator* $\hat{M}\left(k\right)$, Equation (22), in order to encode the addition/subtraction arising on the right-hand side of the Dirac equation through the mass term, cf. Equations (7)–(9), in a deterministic way through suitable permutations. Our finding in this *“Necklace of Necklaces”* model has been that permutations of spins on one necklace are conditioned by a measurement result on the neighboring one. – It appears that all earlier attempts to formulate an automaton for the Dirac equation, to the best of our knowledge, instead had to make use of interfering amplitudes borrowed from quantum mechanics or of probabilistic evolution [29,30,31,32,33,34,35]. In distinction from our classical automaton, these models are cellular automata of a different (nondeterministic) kind. They are known as *quantum cellular automata* and *probabilistic cellular automata*, respectively. See also references [36,37,38,39,40,41] for related more general discussions of probability and quantum theory.

This may all seem rather tedious. However, it illustrates that addition and subtraction on a finite set of adjacent integers with periodic boundary conditions can be obtained simply from permutations of these numbers, which represent discrete automaton states. Such a description is obviously not limited to small sets of such states.

Clearly, since there are non-commuting transpositions (exchange interactions) $\hat{P}$ and operators $\hat{L}$ involved which measure the transverse position of an up spin, it will not be straightforward to extract the overall Hamiltonian governing this automaton. An approximate result can always be obtained with the help of a Baker–Campbell–Hausdorff formula; yet, an exact result would be of interest and we intend to report this elsewhere.

In the end, the discrete integer variables that we have dealt with can be mapped to continuous real ones by invoking *Sampling Theory*, as we recalled in Section 2.1; see [7,25,26,27]. We remind the reader that the value of the wave function in the discrete model is determined at each longitudinal spatial position 2k or 2k+1 by Equations (20) and (21). In this way, letting the necklaces of the model become sufficiently large – i.e., the *separately conserved numbers of up and down Ising spins* – the spinor field is free to move so that its absolute value can possibly become large in some places or small in others in the continuum limit.

## 4. Conclusions

To summarize, a discrete deterministic automaton representation for the Dirac equation in 1 + 1 dimensions is presented, which is based on Equations (10) and (11). In order to incorporate the structure of these finite differences equations, especially the arithmetic required by their right-hand sides, by making use of permutations of two-state spins in an underlying classical Ising model, a torus-like compactification of the state space has been introduced. Here, the real discrete value of the Dirac field is encoded in the position of an up spin on a transverse “necklace” together with its longitudinal spatial position on a chain with periodic boundary conditions—altogether forming a *“Necklace of Necklaces”*.

Generalizing an earlier study of the Weyl equation [24], which features no mass term, the evolution of this automaton is determined by elementary spin exchange operations, building up permutations from transpositions. This is in accordance with the *Cellular Automaton Interpretation of QM* which requires to build discrete deterministic models with dynamics generated by *permutations of ontological states* [4]. Thus, the *“Necklace of Necklaces”* model described in this work *never* leads to quantum mechanical superposition states, it is a classical automaton.

However, we recall that quantum mechanical features enter such models “by mistake” or, rather, mathematical convenience, as discussed extensively earlier [4,23,24,33,41]. Instead of resuming this here, we conclude by listing several conceivable extensions of the model:

- The generalization to more than 1+1 dimensions should be feasible, notwithstanding some necessary bookkeeping efforts.
- The effective Hamiltonian governing the discrete model should be derived and the continuum limit be understood.
- Symmetries and conservation laws of cellular automata are of general interest beyond the present context.
- A field theory of fermions obeying the Pauli principle should be formulated (relating two-state Ising spins to occupation numbers?).
- An external scalar potential can be considered as a spatially varying mass, yet interactions with dynamical gauge fields will require additional considerations.

This may show that there is ample room for future work.

## Figures and Tables

**Figure A1:**
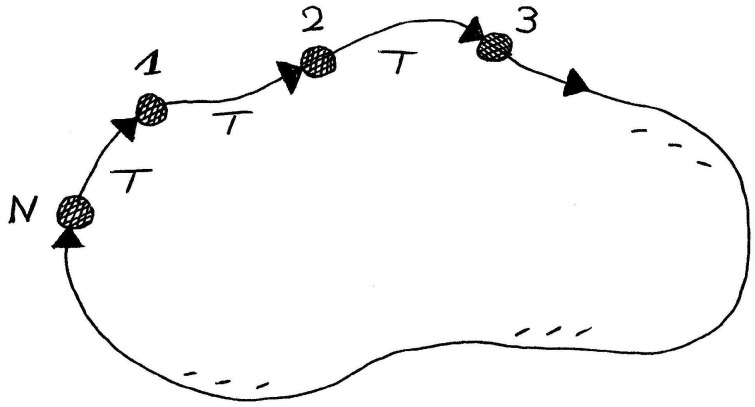
A *Cogwheel* jumps periodically through *N* states, taking time *T* for each jump. *From* [14].

**Figure A3:**
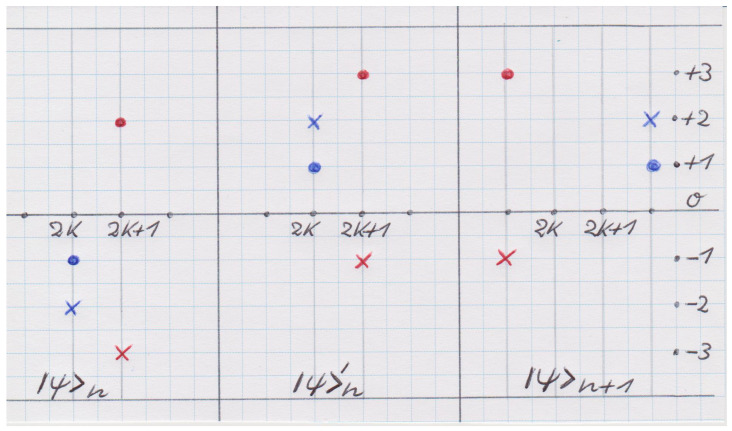
(**Left**): Two initial conditions (heavy dots, crosses) for ${|\Psi \rangle }_{n}$ on two neighboring transverse necklaces attached to the even and odd longitudinal sites 2k (blue) and 2k+1 (red), respectively. Only the transverse positions (drawn vertically, ranging in [−3,+3]) of the single up spins are marked; all others are down. (**Middle**): Update of the initial state by the scattering operator $\hat{M}\left(k\right)$, Equation (22), gives the intermediate result ${|\Psi \rangle }_{n}^{\prime }$. Note that the blue cross has felt the periodic boundary condition in the vertical direction, according to −2+(−3)≡+2. (**Right**): To obtain the final result of the one-step update, ${|\Psi \rangle }_{n+1}$, the up spins at even and odd longitudinal positions have to move two positions to the right and left, respectively.

## Data Availability

The original contributions presented in this study are included in the article. Further inquiries can be directed to the corresponding author.

## Appendix A

### Appendix A.1. Permutations and the Cogwheel Model

The *Cellular Automaton Interpretation of QM* suggests to build discrete deterministic models with dynamics generated by *permutations of ontological states* [4].

The periodicity of such models, without fusion or fission of trajectories in state space, assimilates them to QM models.

Consider *N* objects, $A_{1},A_{2},\ldots ,A_{N}$ (“states”), which are mapped in *N* steps onto one another, involving *all* states. This can be represented by a *unitary* N×N matrix with *one* off-diagonal phase per column and row and with zeroes elsewhere:

(A1) $${\hat{U}}_{N}:=\left(\begin{array}{ccccc} 0 & . & . & 0 & e^{i\phi_{N}}\\ e^{i\phi_{1}} & 0 & \; & . & 0\\ 0 & e^{i\phi_{2}} & . & \; & .\\ . & \; & . & . & .\\ 0 & . & 0 & \;e^{i\phi_{N-1}} & 0 \end{array}\right).$$

The basis of vectors, on which such a *complete permutation* ${\hat{U}}_{N}$ acts, we refer to as the *standard basis*.

Since ${\left({\hat{U}}_{N}\right)}^{N}=e^{i\sum_{k=1}^{N}\phi_{k}}\;1$, the eigenvalues of the corresponding *Hamiltonian* ${\hat{H}}_{N}$, which is defined *via* ${\hat{U}}_{N}=:e^{-i{\hat{H}}_{N}T}$, are obvious. Here, *T* serves to introduce a time scale; its inverse provides an energy scale for the Hamiltonian. (We always use units such that ℏ=c=1.) Thus, we may write down the matrix elements of the Hamiltonian (n=1,…,N):

(A2) $${\left({\hat{H}}_{N}\right)}_{nn}=\mathrm{diag}\left(\frac{1}{NT}\left(2\pi \left(n-1\right)-\sum_{k=1}^{N}\phi_{k}\right)\right),$$

which refer to *diagonal(izing) basis*. Since the arbitrary phases $\phi_{k}$ play no role in what follows, they will be omitted.

The diagonal and standard bases are unitarily related by discrete Fourier transformations. This allows us to calculate the matrix elements of the Hamiltonian with respect to the standard basis:

(A3) $${\left({\hat{H}}_{N}\right)}_{nn}=\frac{\pi }{NT}\left(N-1\right),\;\;n=1,\ldots ,N,$$

and

(A4) $${\left({\hat{H}}_{N}\right)}_{n\neq m}=\frac{\pi }{NT}\left(-1+i\cot\left(\frac{\pi }{N}\left(n-m\right)\right)\right),\;\;n,m=1,\ldots ,N,$$

and ${\left({\hat{H}}_{N}\right)}_{nm}={\left({\hat{H}}_{N}\right)}_{mn}^{*}$, as it should be.

What we have represented here is known as the *Cogwheel Model*; see [4,23] for further details and additional references. With N→∞, T→0, ω:=1/NT fixed, the *Cogwheel Model* can be mapped onto a description of the quantum harmonic oscillator. Such a simple automaton is illustrated in Figure A1.

### Appendix A.2. A Chain of Ising Spins with Exchange Interactions

We consider a chain of 2S classical two-state spins with periodic boundary conditions, which will be always in one of $2^{2S}$ possible *ontological states*. The latter are specified by the two-state variables $s_{k}=\pm 1,\;\;k=1,\ldots ,2S,2S+1$, with $s_{2S+1}\equiv s_{1}$.

Interactions are defined as transpositions of the two-state variables (*spin exchange*) of nearest neighbors:

(A5) $${\hat{P}}_{ij}|s_{i},s_{j}\rangle :=|s_{j},s_{i}\rangle ,\;\;{\hat{P}}_{2S\;2S+1}\equiv {\hat{P}}_{2S\;1},$$

with the following properties of the exchange operators:

(A6) $${\hat{P}}^{2}=1,\;\left[{\hat{P}}_{ij},{\hat{P}}_{jk}\right]\neq 0,\;\;{\hat{P}}_{ij}=\left({\underline{\hat{\sigma }}}_{i}\cdot {\underline{\hat{\sigma }}}_{j}+1\right)/2,$$

where the last equation recalls the relation between exchange operators and vectors formed by the three Pauli matrices, e.g., ${\underline{\hat{\sigma }}}_{k}$ acting on the spin at site *k*; a generalization for larger than two-state spins exists as well.

The dynamics of the chain are then defined by permutations of the chain states which are generated by multiple applications of the exchange operators:

(A7) $$\hat{U}:=\prod_{k=1}^{S}{\hat{P}}_{2k-1\;2k}\prod_{k^{\prime }=1}^{S}{\hat{P}}_{2k^{\prime }\;2k^{\prime }+1}=:\exp\left(-i\hat{H}T\right),$$

indicating once more the definition of the corresponding Hamiltonian. The latter can be obtained analytically as a polynomial in $\hat{U}$ and ${\hat{U}}^{†}$ [23].

We note that, reading the product of transpositions in Equation (A7) from the right to the left, first, all even transpositions are performed and then all odd ones, which commute among each other, respectively—here, even/odd refers to whether the first index *k* in ${\hat{P}}_{k<l}$ is even/odd. This separation of the even/odd transpositions implements a finite signal velocity in the model, such that a perturbation of a particular spin reaches only its nearest neighbors in one update step, which is effected by applying the unitary $\hat{U}$ once on the state of the chain.

The evolution of an arbitrary initial state of the spin chain is very simple and obeys a number of conservation laws [23]. Writing down explicitly a few update steps for a short chain, it can be seen as a composition of *Cogwheel* like motions of *left-movers* and *right-movers*, concerning the spin variables on the *odd* and *even* chain sites, respectively. Recalling the periodic boundary condition on the chain, we obtain ${\left(\hat{U}\right)}^{S}=1$ for a chain of 2S spins. The behavior of this cellular automaton is illustrated in Figure A2 (top).

In order to arrive at a continuum theory of the automaton which leads to the Weyl equation, we need to have access to the arithmetic of variables that are allowed to assume sufficiently large or small integer values, rather than the elementary two-state spins with $s_{k}=\pm 1$ employed so far. It is useful to introduce site *occupation numbers*:

(A8) $$n_{k}:=\left(s_{k}+1\right)/2\;\left(=0,1\right).$$

These can be thought of as eigenvalues of a fermionic number operator $\hat{n}\left(k\right)$, which might be of interest later. They preserve the feature of variables associated with even/odd sites to be right-moving/left-moving, respectively, under the updating affected by $\hat{U}$. Furthermore, they inherit the periodic boundary condition.

Next, we perform an *invertible linear transformation* on the set of occupation numbers defined by:

(A9) $${\tilde{n}}_{k}:=n_{k}+n_{k+2},\;k=1,\ldots ,2s,$$

cf. Figure A2 (bottom). Iterating this transformation a sufficiently large number of *r* times we arrive at *block variables* ${\tilde{n}}^{\left(r\right)}$ that range between zero and $2^{r}$. Again, they move left/right under $\hat{U}$ from an odd/even site, with periodic boundary conditions.

We remark that the block variables must be distinguished from coarse-grained variables that would be obtained by some decimation of the number of degrees of freedom.

### Appendix A.3. Illustration of Scattering Operator $\hat{M}$ and Automaton Update

In Figure A3 we illustrate by two examples how the *scattering operator* $\hat{M}\left(k\right)$, defined in Equation (22), acts in the update process of the automaton.

We have shown here an automaton that is very small (with periodic boundary conditions) in the transverse direction for simplicity. Eventually, a numerical simulation of consecutive updates would be amusing to see for a large automaton—one that follows the deterministic rules constructed from the Dirac equation in this work.
